# Tracking the contamination sources of microbial population and characterizing *Listeria monocytogenes* in a chicken slaughterhouse by using culture-dependent and -independent methods

**DOI:** 10.3389/fmicb.2023.1282961

**Published:** 2023-11-30

**Authors:** Jiyeon Jeong, Hyokeun Song, Woo-Hyun Kim, Myeongju Chae, Ji-Youn Lee, Yong-Kuk Kwon, Seongbeom Cho

**Affiliations:** ^1^Avian Disease Research Division, Animal and Plant Quarantine Agency, Gimcheon-si, Gyeongsangbuk-do, Republic of Korea; ^2^College of Veterinary Medicine and Research Institute for Veterinary Science, Seoul National University, Seoul, Republic of Korea

**Keywords:** *Listeria monocytogenes*, chicken slaughterhouse, multi-locus sequence typing, real-time quantitative polymerase chain reaction, 16S rRNA gene amplicon sequencing, source tracking, culture-dependent method, culture-independent method

## Abstract

*Listeria monocytogenes* is the etiologic agent of listeriosis, a foodborne disease that poses a threat to public health globally. Chicken meat exhibits heightened susceptibility to *L. monocytogenes* contamination during butchery. The persistence of this pathogen in the slaughterhouse environment enables recurring contamination of meat products. This study aimed at identifying the sources and transmission routes of *L. monocytogenes* contamination within an abattoir where it was consistently detected for three consecutive years (2019–2021). Furthermore, the environmental factors aiding contamination along chicken processing lines were determined by surveying the microbiome within the facility. Samples collected in 2019 to 2021 were subjected to culture-dependent analysis to assess the prevalence, serotypes, and multi-locus sequence typing (MLST) of *L. monocytogenes*. Additionally, the specimens collected in 2021 underwent culture-independent analysis via real-time quantitative polymerase chain reaction (qPCR) and 16S rRNA gene amplicon sequencing to identify the contamination sources and characterize the entire microbial community within the slaughterhouse. *L. monocytogenes* was isolated only from the clean zone, where the final slaughtering stage occurs. Most strains isolated from the final carcasses showed the same genetic cluster as the isolate in the chilling water and were assigned to MLST profile ST3. Culture-independent qPCR confirmed *L. monocytogenes* contamination in all samples, excluding post-scalding carcasses, prewashed post-evisceration carcasses, and the bleeding areas. Consequently, qPCR enabled more comprehensive identification of *L. monocytogenes* contamination points than culture-dependent approaches. Moreover, 16S rRNA gene amplicon sequencing demonstrated that psychro-tolerant and spoilage-related bacteria with *L. monocytogenes*-like attributes exhibited enhanced viability in the clean zone and immersion-chilling water. Metagenomics-based source tracking analysis further revealed that the shackles and chilling waters represent predominant sources of cross-contamination between different slaughterhouse zones, whereas the grading and packaging workstations and chilling water in the clean zone were deemed crucial sources affecting final carcass contamination. Collectively, these findings demonstrate through culture-dependent and -independent methods that *L. monocytogenes* spreads along the slaughter line, contaminating the slaughterhouse. Moreover, by investigating changes in microbial community and bacterial flow along the slaughter line within the facility, the sources influencing carcass contamination can be effectively traced.

## 1 Introduction

*Listeria monocytogenes*, a foodborne pathogen, is widespread in the environment and livestock (Buchanan et al., 2017). The bacterium contaminates various food products, including vegetables, milk, dairy products, poultry, and meat products (Ryser et al., 2007). Although listeriosis is relatively uncommon compared to salmonellosis or campylobacteriosis, it is a potentially fatal disease (Goh et al., 2014). Notably, based on reports from the European Food Safety Authority (EFSA), listeriosis is the most common cause of death from foodborne illness in the European Union and one of the most serious foodborne diseases under EU surveillance [European Food Safety Authority and European Centre for Disease Prevention and Control (EFSA and ECDC), 2018, 2022]. Furthermore, case–control studies conducted in the United Kingdom, United States, and Canada during the 1980s, 2004, 2008, and 2019 have consistently demonstrated a significant association between listeriosis and consumption of cooked chicken, chicken wraps, and undercooked chicken (Schwartz et al., 1988; Marcus et al., 2009; Little et al., 2012; McLauchlin et al., 2020).

Chicken meat slaughterhouses and processing facilities are common sites for *L. monocytogenes* growth and proliferation. Therefore, effective control of this hazard becomes a significant challenge for poultry-processing companies to prevent economic losses and public health risks associated with product recalls or condemnation by veterinary inspection services (Rørvik et al., 2003; Sakaridis et al., 2011; Moura et al., 2016; Oliveira et al., 2018; Iannetti et al., 2020). Certain *L. monocytogenes* strains can persist in slaughterhouses and may repeatedly contaminate meat products. Therefore, understanding the specific processing steps in which *L. monocytogenes* contamination occurs is crucial for appropriate control measures within slaughterhouses.

The gold standard test for detecting *L. monocytogenes* is the culture technique performed per the ISO 11290-1 protocol (International Organization for Standardization, 2017; Jandaghi et al., 2020); however, in the culture-dependent method, the bacterium may not be isolated owing to the sample state, culture conditions, or the presence of competing organisms. Hence, studies using culture-independent methods are needed to identify the points and sources of *L. monocytogenes* contamination within the slaughterhouse, to supplement the culture-dependent results. Indeed, molecular methods are being used in the food industry. In particular, real-time quantitative polymerase chain reaction (qPCR) offers higher sensitivity than other methods and can be used to quantify foodborne pathogens (Shi et al., 2010; Heo et al., 2014; Liu et al., 2019; Bai et al., 2022). For example, qPCR has been used to detect foodborne pathogens in the environment and carcasses in slaughterhouses to supplement false-negative results obtained via culture methods. Moura et al. (2019) compared the detection results of *L. monocytogenes* in carcass and environmental samples of a slaughterhouse using the standard culture method and qPCR and recommended their combined use. In addition, next-generation sequencing is also utilized to identify the main contamination points in slaughterhouses by characterizing complex microbial communities and population flow (Zwirzitz et al., 2020; Chen et al., 2020a,b). Additionally, metataxonomic analysis may provide in-depth insights into the slaughterhouse environment. Hence, these strategies should be adopted to characterize *L. monocytogenes* isolates in slaughterhouses, facilitating the development of appropriate strategies to control their contamination.

Accordingly, the primary of the current study is to identify the sources and transmission routes of *L. monocytogenes* contamination in a slaughterhouse where *L. monocytogenes* has been consistently detected for three years and define the microbial ecology and dynamics along the slaughter line. To this end, we first investigated the prevalence, serotypes, and multi-locus sequence typing (MLST) of *L. monocytogenes* isolated from the slaughterhouse using culture methods. We then identified the points of *L. monocytogenes* contamination by supplementing the false-negative results obtained via culture methods through qPCR. Finally, to understand how the slaughterhouse environment affects carcass contamination, we analyzed the microbial community and bacterial flow of carcass and environmental samples at each slaughtering step using 16S rRNA gene amplicon sequencing.

## 2 Materials and methods

### 2.1 Description of facility and sampling

**Figure 1:**
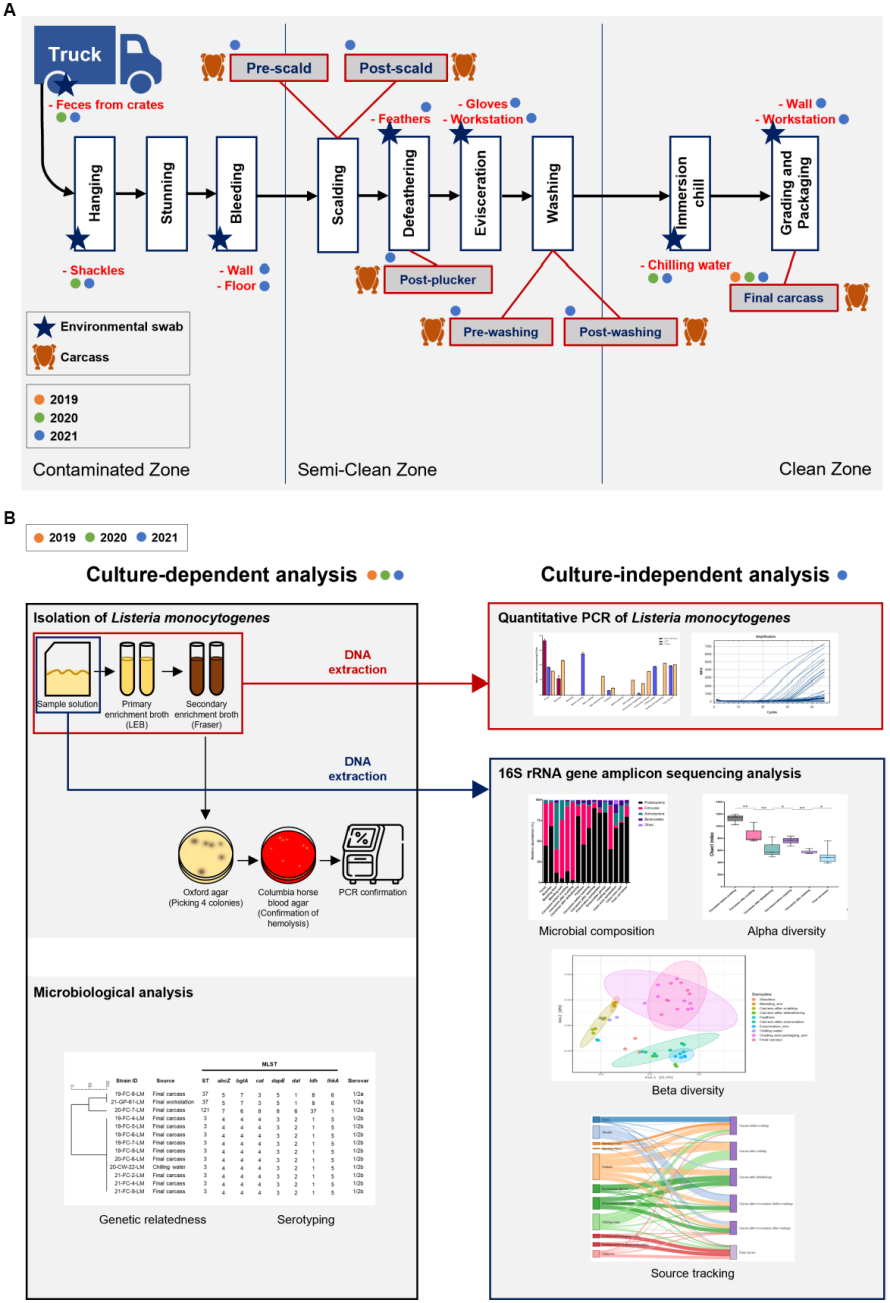
**(A)** Chicken processing line and the stages of sample collection. Steps of collecting carcass and environmental samples are indicated by chicken and star shapes, respectively, and samples collected by year are marked with orange (2019), green (2020), and blue (2021) circles. **(B)** Schematic of the present study. All samples from 2019, 2020, and 2021 were analyzed for serotypes and genetic relatedness by using culture-dependent methods. For samples of 2021, additional culture-independent analysis was conducted to identify the contamination points of *Listeria monocytogenes*, and the microbial community throughout the slaughterhouse was described.

Samples were collected from a slaughterhouse located in Chungcheongnam-do Province, Republic of Korea. Characteristics of the surveyed abattoir are listed in Supplementary File 1. We visited this facility once every summer (August or September) from 2019 to 2021. Ten carcasses were sampled after immersion chilling (final carcasses) in 2019. Ten final carcasses and environmental samples (feces, shackles, and chilling water) were collected in 2020. Additionally, in 2021, carcasses from six processing steps and environmental samples from ten sites throughout the abattoir were collected. Chicken carcasses were collected at six processing steps, including pre-scald (*n* = 3), post-scald (*n* = 3), post-plucker (*n* = 3), prewashing after evisceration (*n* = 3), post-washing after evisceration (*n* = 3), and post-immersion chill (final carcass; *n* = 10). The environmental samples comprised feces swabs of truck crates containing live birds (*n* = 5), swabs of shackles (*n* = 5), surface swabs of the walls (*n* = 3) and floors (*n* = 3) in the bleeding area, feathers of the plucking machine (*n* = 5), surface swabs of evisceration workers’ gloves (*n* = 5) and workstation (*n* = 5), 400 mL of chilling water (*n* = 3), and surface swabs of the grading and packaging workstation (*n* = 3) and walls (*n* = 3) (Figure 1A). Figure 1A illustrates the slaughtering stages of the production chain and sampling positions each year. After selecting one chicken flock, sampling was performed along the slaughter line from the time the flock’s truck entered the facility until slaughtering was complete. Each carcass was placed in a poultry rinse bag (37.5 × 50 cm; Nasco, WI, United States), and 400 mL of buffered peptone water (BPW; BD Biosciences, CA, USA) was poured into the rinse bag. The carcasses were manually agitated for 2 min to ensure thorough contact of BPW with the surface and internal parts of the chickens. The carcass rinsate was transferred into a sterile sampling bottle and stored in a cool box until sampling was complete. The environmental swab samples were rubbed with a Speci-sponge (Nasco) by wiping horizontally for approximately ten times, flipped, and then wiped vertically. Feathers were collected and put into a Whirl-pack bag (Nasco). Chilling water was poured from an immersion water chiller tank into a sterile bottle. All samples were transported to the laboratory in an icebox and processed on the same day.

### 2.2 Sample preparation and DNA extraction for metagenomic analysis

The study design for culture-dependent and -independent analyses is illustrated in Figure 1B. From samples collected in 2019 and 2020, *L. monocytogenes* was isolated for culture-dependent analysis to determine the prevalence, serotypes, and sequencing types (STs). Samples collected in 2021 were processed for culture-dependent and -independent analyses to detect the contamination points and describe the whole microbial community in the slaughterhouse (Figure 1B). For swab samples of sponges and feathers, 20 mL BPW was added into each Speci-sponge and Whirl-pack bag containing feathers. Each sponge and sample bag were homogenized for 2 min, and the suspension was transferred into a 50-mL conical tube (Corning Life Sciences, NY, United States) for subsequent analyses.

To perform qPCR and 16S rRNA gene amplicon sequencing, DNA was extracted from sample solutions and primary and secondary enrichment cultures using a DNeasy Powersoil Pro Kit (Qiagen, Hilden, Germany) in accordance with the manufacturer’s instructions (Figure 1B). The quantity and quality of DNA were measured using a NanoDrop spectrometer (Thermo Scientific, Wilmington, DE, United States).

### 2.3 Isolation of *Listeria monocytogenes* and microbial analysis

*L. monocytogenes* was isolated in accordance with the Korean Food Standards Codex manual.^1^ Swab and feather suspension (1 mL each) and 25 mL of carcass rinsate were inoculated into 9 mL and 225 mL of Listeria Enrichment broth (LEB; BD Biosciences), respectively. In addition, 25 mL of chilling water was added to 225 mL of LEB. They were incubated at 30°C for 24 h and transferred to Fraser broth (BD Biosciences). The cultures were incubated 37°C for 48 h, streaked onto Oxford agar (BD Biosciences), and incubated again at 37°C for 48 h. Up to four typical *L. monocytogenes* colonies were cultivated on blood agar containing 5% (v/v) defibrillated sheep blood (Synergy Innovation, Seongnam-si, Republic of Korea) and incubated at 37°C for 24 h. Typical colonies showing β-hemolysis were identified using VITEK2 Gram-Positive Identification (GPI) cards (bioMérieux, NC, United States) following the manufacturer’s instructions and through amplification of *hlyA* and *iap* by PCR as previously described (Chen et al., 2017).

For microbiological analysis, serotyping and MLST analysis were performed on *L. monocytogenes* strains (2019, *n* = 6; 2020, *n* = 3; and 2021, *n* = 4) (Supplementary File 2). Serotyping of *L. monocytogenes* was performed by a slide agglutination assay using commercial Listeria antisera (Denka Seiken, Tokyo, Japan) in accordance with the manufacturer’s instructions. MLST analysis based on seven housekeeping genes (*abcZ*, *bglA*, *cat*, *dapE*, *dat*, *ldh*, and *lhkA*) was performed on all isolates using primers and protocols described in the Institut Pasteur MLST database^2^ (Moura et al., 2016). A dendrogram for visualizing genomic relationships among the isolates was generated using BioNumerics v.8.0 (Applied Maths, Sint-Martens-Latem, Belgium).

### 2.4 Culture-independent analysis

#### 2.4.1 qPCR of *Listeria monocytogenes*

qPCR was conducted using DNA extracted from 65 samples, including sample solutions and primary enrichment samples, and 58 secondary enrichment samples suspected to be positive as the broth turned black (Figure 1B). *prfA* was selected as the target gene for qPCR, and primers and probes were used as previously described (Jandaghi et al., 2020). All reactions were carried out in a final volume of 20 μL comprising 3 μL of template DNA, 1 μL of each primer (10 pmol/μL of each primer), 0.5 μL probe (10 pmol/μL), 10 μL IQ supermix (Bio-Rad, CA, United States), and 4.5 μL distilled water. The amplification conditions were 94°C for 5 min, followed by 45 cycles for 25 s at 94°C, and 20 s at 51°C. Distilled water (3 μL) was used as the negative control, and all reactions were run in triplicate using a CFX96 35 Real Time system C1000 Touch thermal cycler (Bio-Rad). Standard curves were generated by qPCR using serial dilutions of DNA extracted from ATCC 19115 through the same method. The copy numbers of *prfA* of DNA standards were defined as previously described (Jandaghi et al., 2020), and those of DNA samples were calculated based on the linear regression of the logarithmic values of standards and qPCR cycle threshold (Cq) values.

#### 2.4.2 Sequencing of 16S rRNA gene amplicon

Library preparation and sequencing were performed by ChunLab (Seoul, Republic of Korea). Briefly, 16S rRNA gene (V3–V4) was amplified using TaKaRa Ex Taq DNA polymerase (TaKaRa, Kyoto, Japan) and primers (341F 5′-TCGTCGGCAGCGTCAGATGTGTATAAGAGACAGCCTAC GGGNGGCWGCAG-3′ and 805R 5′-GTCTCGTGGGCTCGGAGATGTGTATAAGAGACAGGAC TACHVGGGTATCTAATCC-3′) for library preparation. Barcodes were added during the second round of amplification with i5 forward primer and i7 reverse primer. Next, the amplified PCR products were purified using CleanPCR (CleanNA, Waddinxveen, Netherlands) and pooled together; short fragments were removed using CleanPCR (CleanNA). Sequencing was performed using an MiSeq platform (Illumina, San Diego, CA, United States) and an MiSeq Reagent Kit v2 (500 cycles).

#### 2.4.3 Processing and analysis of sequencing data

Raw sequences were analyzed using the microbiome taxonomic profiling pipeline in EzBioCloud (ChunLab).^3^ Paired-end reads were filtered by quality (*Q*-value <25) and merged using PANDAseq software. Primer trimming and denoising were performed using an in-house program of ChunLab. The taxonomic assignment of sequences was performed using USEARCH (Edgar, 2010) with a 97% similarity cut-off for species-level identification from the EzBioCloud 16S database and then clustered, and operational taxonomic units (OTUs) were defined.

The composition and bacterial diversity of the microbial community were analyzed using CLCommunity software (ChunLab) and visualized using the R project ggplot2 (R software v.4.2.0). A heatmap was generated using the Heatmap 2 function of the R ggplot2 package. The alpha diversity of the microbial community was measured using CLCommunity software (ChunLab) including the Simpson’s and Shannon’s indices, representing richness and evenness, respectively. Differences in alpha diversity between sample groups were evaluated using the Mann–Whitney *U*-test. Beta diversity was determined using principle coordinate analysis (PCoA) plots of Bray–Curtis distance through the “phyloseq” R-package (McMurdie and Holmes, 2013) and evaluated using the analysis of group similarities test. Microbial sources were tracked using a source-tracker model based on Bayesian algorithm.^4^ Environmental samples from the facility were assigned as sources, and the processing and final carcass samples were assigned as sinks. The source environment proportions for meat samples and each flow between the source and sink were visualized as Sankey flow diagrams using R (v.4.2.0).

## 3 Results

### 3.1 Isolation, serotyping, and genetic relatedness analysis based on MLST analysis of *Listeria monocytogenes*

In 2019, *L. monocytogenes* was isolated from six out of ten final carcasses (60.0%). In 2020, it was isolated from three (13.0%) samples, including two final carcasses and one chilling water sample, out of 23 samples, comprising ten final carcasses, five feces samples from crates, and three chilling water samples. Among the 65 samples collected in 2021, comprising 15 intermediate-stage carcasses during slaughtering, 10 final carcasses, and 40 environmental samples, *L. monocytogenes* was isolated from three final carcasses and one surface sample from the grading and packaging workstation (6.2%) (Supplementary File 3). The majority of *L. monocytogenes* was isolated from the final carcasses. In the slaughterhouse environment, the bacteria were found on the surface of the grading and packaging workstation and in chilling water. All isolates were identified within the clean zone of the sites.

**Figure 2:**
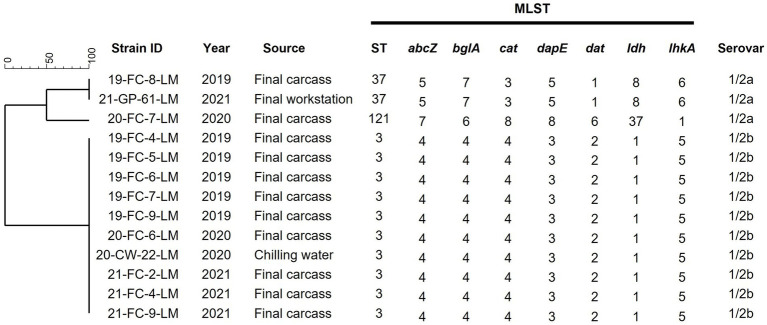
Serotypes and MLST analysis of 13 *Listeria monocytogenes* isolates. Phylogenetic tree was based on the seven concatenated gene sequences. Final carcass, carcass after immersion chilling; Final workstation, surface of the grading and packaging workstation; ST, sequence type.

Thirteen *L. monocytogenes* strains isolated over three years were analyzed and characterized. The serovars and STs of the isolates are shown in Figure 2. The predominant serotypes among all strains were 1/2b, isolated from ten final carcasses collected between 2019 and 2021 and one chilling water sample of 2020. Genetic relatedness results obtained via MLST analysis revealed that the strains were classified into three distinct STs: ST3 (76.9%, 10/13), ST37 (15.4%, 2/13), and ST121 (7.7%, 1/13). Most strains belonged to ST3, with nine isolated from final carcasses and one from a chilling water sample. Notably, the ST3 strains were consistently isolated from the final carcasses over the three-year period, forming an identical genetic cluster.

### 3.2 Overall contamination by *Listeria monocytogenes* across the slaughterhouse determined By qPCR

**Figure 3:**
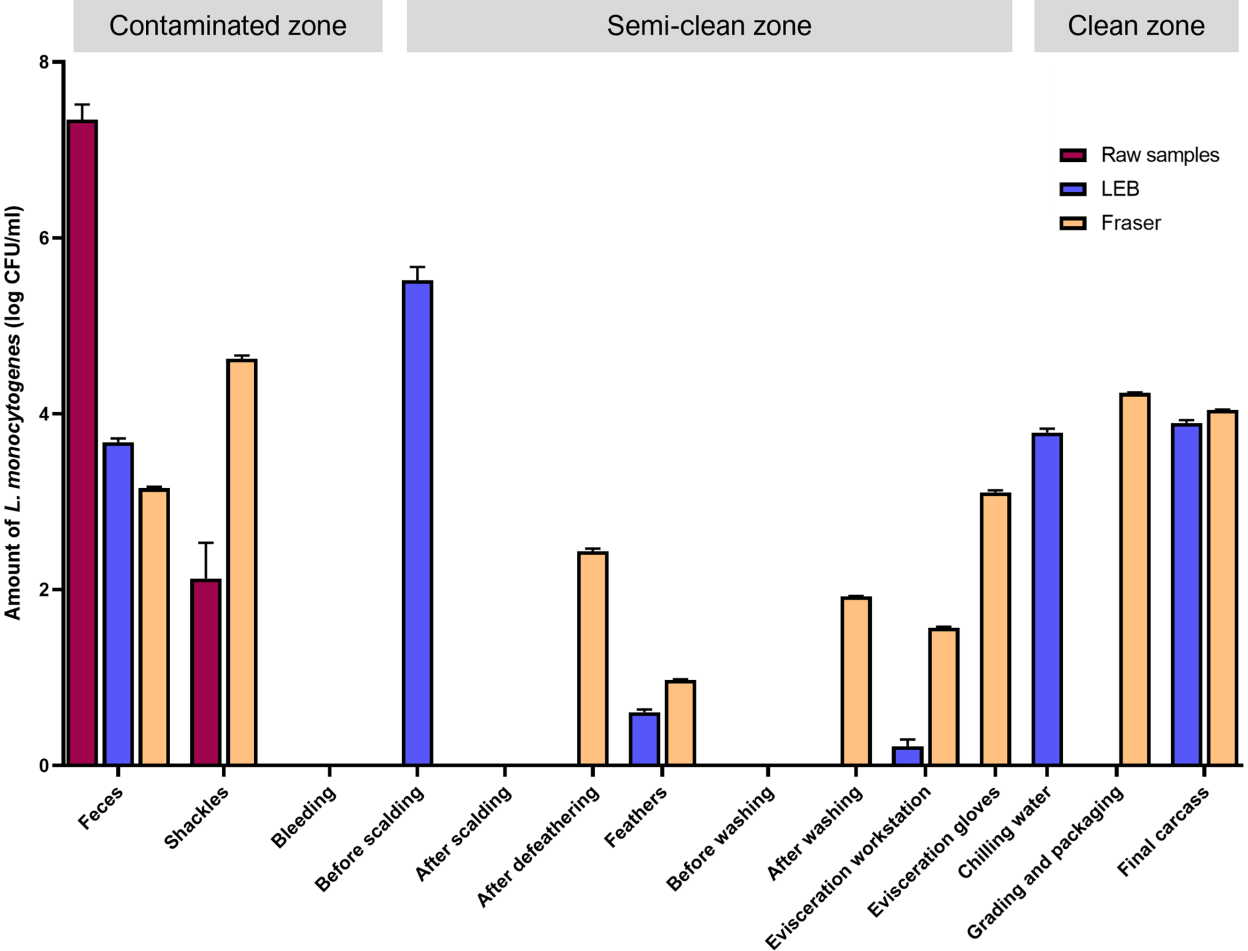
Quantitative PCR for *Listeria monocytogenes* by using raw, primary enrichment, and secondary enrichment samples. Log CFU/mL of *L. monocytogenes* in each sampling site inferred from the standard curve. Raw samples, carcass rinsate or sample solution; LEB, sample cultures in *Listeria* enrichment broth (primary enrichment broth of *L. monocytogenes*); Fraser, sample cultures in Fraser broth (secondary enrichment broth of *L. monocytogenes*).

The *L. monocytogenes* population at the sampling sites was estimated by qPCR targeting *prfA* (Figure 3). The log colony-forming units (CFU)/mL of *L. monocytogenes* in each sample solution (without enrichment) were 7.34 ± 0.17 [mean ± standard error of mean (SEM)] for feces from crates and 2.13 ± 0.4 (mean ± standard error of mean) for shackle swabs. *L. monocytogenes* was not detected in any other sample solutions (without enrichment). However, in enriched LEB cultures (primary enrichment), contamination by *L. monocytogenes* was detected in various samples. The log CFU/mL were as follows: feces (3.68 ± 0.04), carcasses before scalding (5.52 ± 0.15), feathers from plucking machines (0.61 ± 0.03), surface of the evisceration workstation (0.22 ± 0.08), chilling water (3.79 ± 0.04), and final carcasses (3.90 ± 0.03). In Fraser broth cultures (secondary enrichments), *L. monocytogenes* was detected in feces (3.15 ± 0.02), shackles swabs (4.63 ± 0.03), carcasses after defeathering (2.44 ± 0.03), feathers (0.97 ± 0.01), carcasses after evisceration and washing (1.92 ± 0.01), surface of the evisceration workstation (1.57 ± 0.01), evisceration workers’ gloves (3.11 ± 0.02), surface of the grading and packaging area (4.24 ± 0.01), and final carcasses (4.04 ± 0.01) (Figure 3). Overall, excluding the surface of the bleeding area, carcasses after scalding, and prewashed carcasses after evisceration, all carcass and environmental samples were contaminated with *L. monocytogenes*. Similar to the culture method results, approximately 4 log CFU/mL *L. monocytogenes* was detected in the samples collected from washing to the final slaughtering stage, as observed in enriched LEB and Fraser broth cultures (Supplementary File 4).

### 3.3 Differences in microbial composition at each step of the slaughterhouse determined by 16S rRNA gene amplicon sequencing

**Figure 4:**
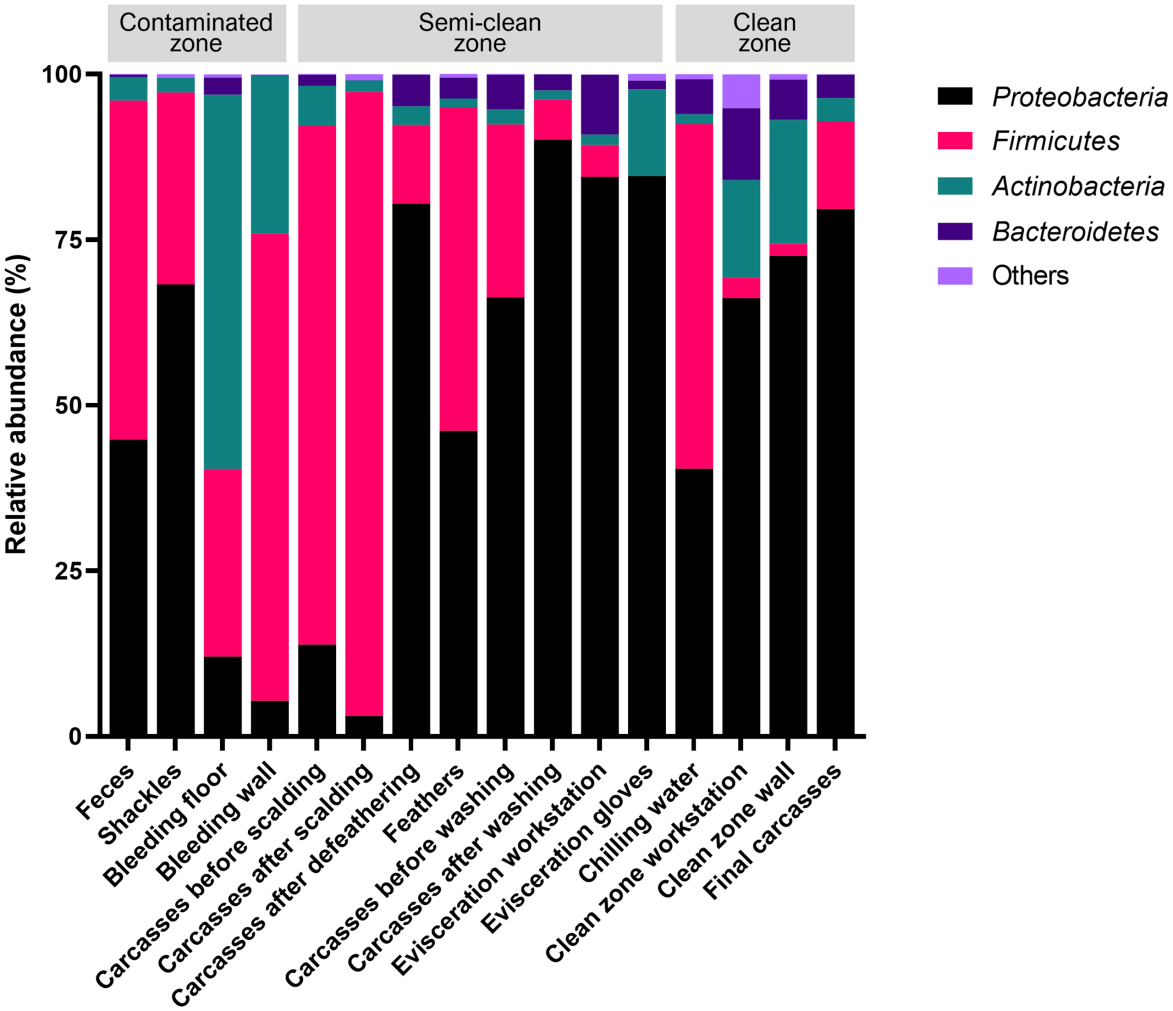
Relative abundances of the four most abundant phyla across all sampling points.

**Figure 5:**
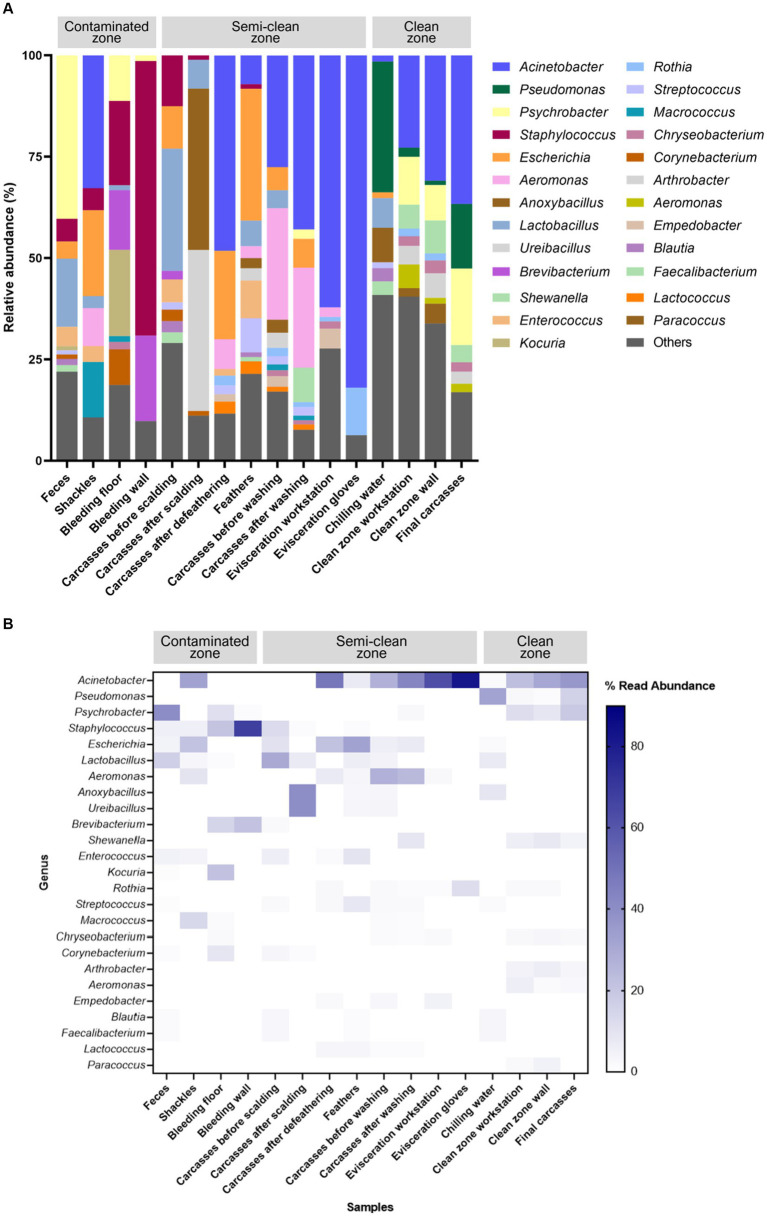
**(A)** Taxonomy bar plot and **(B)** heatmap of the 25 most abundant genera. The *X*-axis and *Y*-axis represent samples along the slaughter line and 25 taxa, respectively. The color scale represents the relative abundance of taxa in individual samples.

The V3–V4 region of the 16S rRNA gene was sequenced for microbiome analysis. A total of 2,852,500 sequencing reads were generated from 50 samples, ranging from 12,627 to 138,750 reads per sample. In total, 4,853 OTUs were identified in all samples. The dominant phyla, with an average abundance >1%, were Proteobacteria, Firmicutes, Actinobacteria, and Bacteroidetes. Proteobacteria and Firmicutes were the predominant phyla in the carcass and environmental samples, excluding the floor surface samples in the bleeding area, which predominantly contained Actinobacteria (56.54%), although they presented diverse microbial communities at each sampling point (Supplementary File 5 and Figure 4). To display these differences on a fine scale, the 25 most abundant genera across all samples were presented using bar plot and heatmap (Figure 5). At the genus level, *Acinetobacter* showed the highest relative abundance across all samples and was highly abundant in the carcass (prewashing after evisceration, 27.54%; post-washing after evisceration, 42.93%) and environmental (workstation, 62.19%; gloves, 81.97%) samples during evisceration. Additionally, a high abundance of *Acinetobacter* was detected in the samples from the clean zone (grading and packaging workstation, 22.75%; walls, 30.91%; and final carcasses, 36.62%). Contamination by *Acinetobacter* was high from the evisceration to the final stage. *Psychrobacter* and *Pseudomonas*, following *Acinetobacter*, were the most relatively abundant genera in the environmental and carcass samples of the final stage in the clean zone. Particularly, *Pseudomonas* was not present in the early slaughtering stages but represented the most prominent genera in the chilling water (32.26%) and was the third most abundant genus in the final carcasses (15.97%), following *Acinetobacter* (36.62%) and *Psychrobacter* (18.87%). By contrast, *Staphylococcus* was highly abundant in the contaminated zone but was not detected in the clean zone environment or in the final carcasses.

### 3.4 Microbial community structure changes along the slaughter line

**Figure 6:**
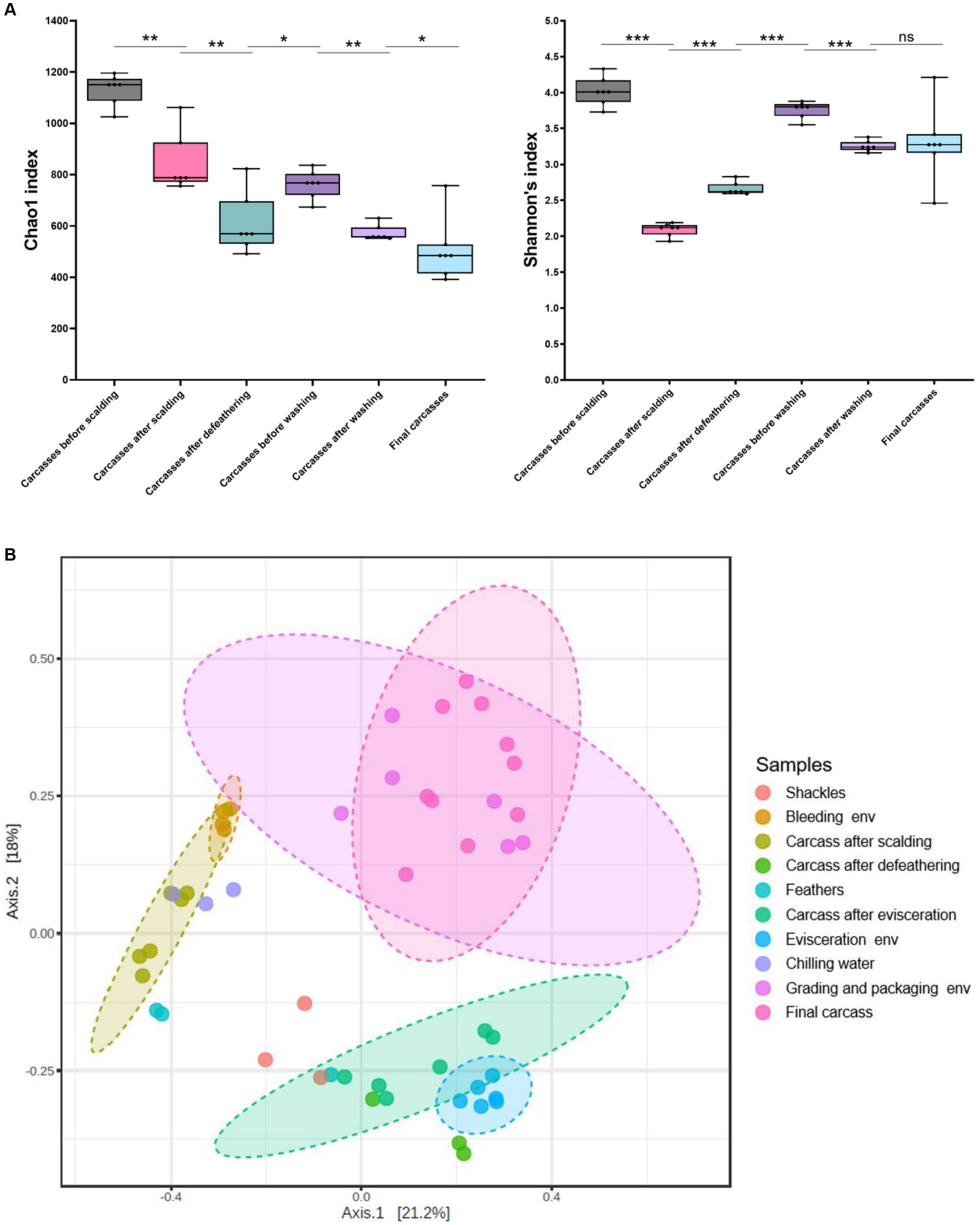
**(A)** Alpha diversity indices (Chao1 and Shannon) of carcass samples along the slaughter line. Boxes indicate the interquartile range (75th–25th) of the data. The line inside each box represents the median value, and the upper and bottom lines are high and low values, respectively. Levels of significance: ns, *p* > 0.05; *p ≤ 0.05; **p ≤ 0.01; ***p ≤ 0.001. **(B)** PCoA plot of Bray–Curtis distances based on 16S rRNA gene libraries obtained from carcass and environmental samples. Colors of each point represent different sampling sites as shown in the legend. Ellipses indicate 95% confidence intervals.

We estimated microbial diversity within carcass samples using alpha diversity indices (Chao1 and Shannon indices). When comparing the six carcass sample groups using the Mann–Whitney U-test, we observed a significant overall decrease in species richness (Chao1) from the beginning to the end of the slaughter line (*p* ≤ 0.05), excluding the carcasses after defeathering and before washing, a significant increase in richness was observed (*p* ≤ 0.05) (Figure 6A). The Shannon index indicated that species diversity was lowest in the carcasses after scalding. By contrast, the diversity was significantly higher in the carcasses after defeathering and before washing than in those after scalding (*p* ≤ 0.001) and defeathering (*p* ≤ 0.001) (Figure 6A). Additionally, the diversity was significantly lower in the carcasses after washing than before washing (*p* ≤ 0.001). However, no significant difference in diversity was observed between the carcasses after washing and the final carcasses (*p* > 0.05) (Figure 6A). Correlations between sampling sites are shown in Figure 6B to understand the impact of processing interventions on microbial diversity. Beta diversity, determined by PCoA based on the Bray–Curtis dissimilarity, indicated that the microbial communities in the samples showed similar patterns based on the sampling position. Specifically, except for chilling water samples, the microbial communities of the environmental and carcass samples from the clean zone showed overlapping patterns distinct from that of the other samples.

### 3.5 Distinct transmission routes throughout the slaughterhouse determined by source tracking analysis

**Figure 7:**
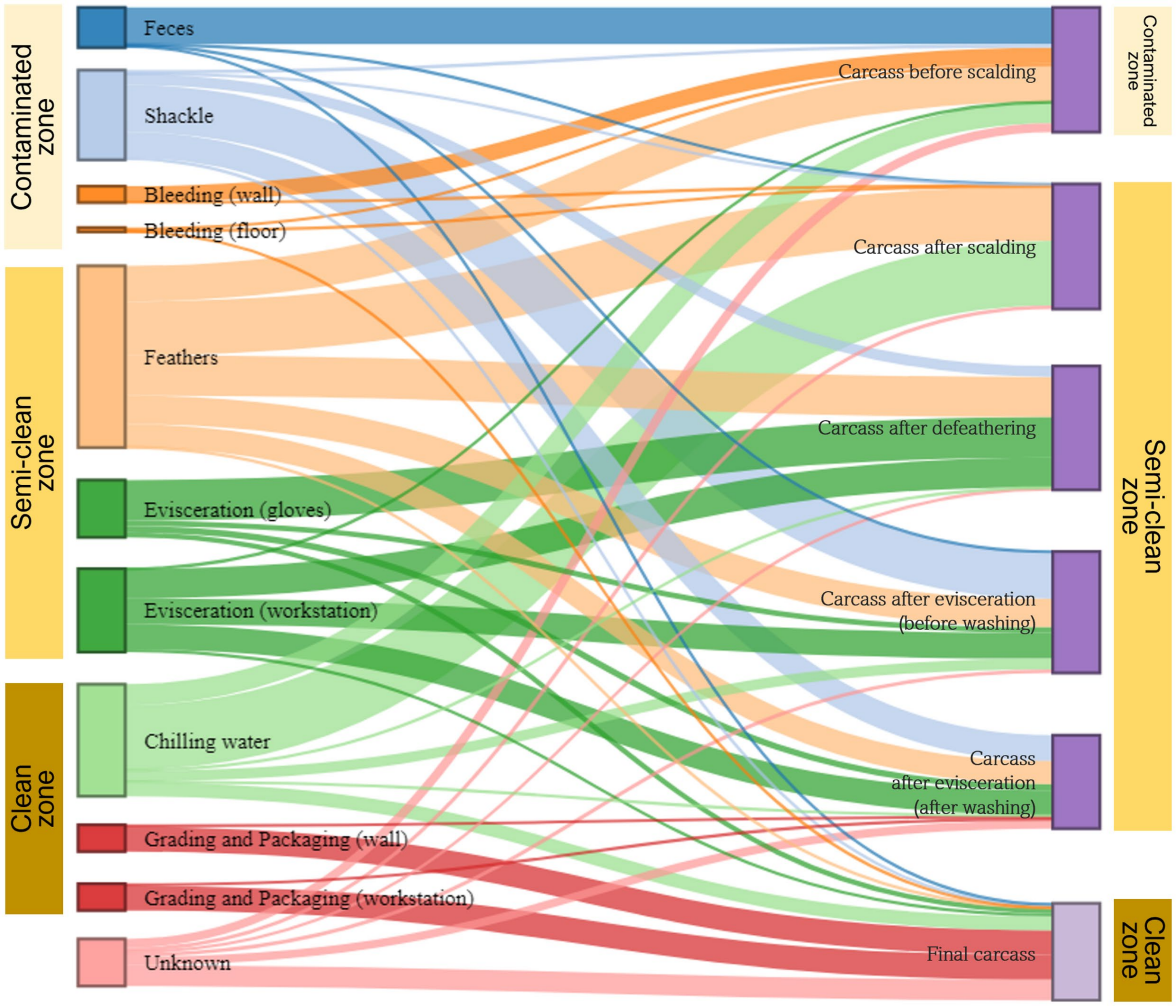
Microbial source tracking for carcass samples by slaughter zone. The Sankey flow diagram illustrates the relative influence of environmental samples (sources) on the carcass samples (sinks). The sources and sinks are shown on the left and right sides, respectively. In the source tracking analysis, “unknown source” refers to a microbiome present in the carcass samples but not detected in the source samples we collected. The width of each flow line between sources and sinks represents the proportion of microorganisms from source samples to those of sink samples. The sum of heights of the bars in the sink samples on the right (carcasses before scalding to the final carcasses) is 100%. On the left, the height of each bar in source samples represents the sum of proportions to each of the six samples.

We used the SourceTracker model to estimate the transfer of microorganisms from environmental to carcass samples along the slaughter line. Given the microbial flow in each of the three zones (contaminated, semi-clean, and clean) of the slaughterhouse, bacteria from sources other than shackles and chilling water were primarily associated with the carcasses (sink samples) in the representative zone from which they were collected. By contrast, bacteria from shackles in the contaminated zone were largely linked with the carcasses in the semi-clean zone, and bacteria from chilling water in the clean zone were associated with the carcasses in all zones (Figure 7). The microorganisms in feces were identified as the main source of contamination in the carcasses before scalding. Shackle samples greatly contributed to the microbial community of the evisceration carcass samples before and after washing, with 37.3 and 20.7%, respectively. However, their contribution substantially decreased to approximately 1.8% in the final carcasses. Feathers played a crucial role in the microbial community of all carcass samples except the final carcasses, particularly in those after scalding (43.0%) and defeathering (32.0%). Most of the microorganisms in the final carcasses were associated with three sources: workstation (41.9%) and walls (19.6%) of grading and packaging, and chilling water (11.2%). Notably, approximately 61.4% bacteria in the final carcasses were associated with the grading and packaging environmental samples. Beta diversity analysis indicated an overlapping clustering pattern between the grading and packaging samples and the final carcasses, suggesting that these sample groups shared similar species. Source tracking analysis further validated that the grading and packaging environmental samples were a significant source of contamination for the final carcasses (Figure 7).

## 4 Discussion

*L. monocytogenes* is a critical foodborne pathogen that raises public health concerns. Several countries, including the Republic of Korea, have implemented a zero-tolerance policy for *L. monocytogenes* in ready-to-eat meat and poultry products (Shank et al., 1996; Baek et al., 2000). The environment of a meat-processing facility with favorable conditions, such as temperature and humidity, poses a significant risk regarding *L. monocytogenes* contamination (Moura et al., 2016). This bacterium can thrive on various surfaces, including floors, drains, countertops, utensils, and equipment. Consequently, it can persist for months or years, leading to recurrent food contamination (Markkula et al., 2005; Carpentier and Cerf, 2011).

In this study, *L. monocytogenes* was isolated from chicken carcasses and slaughterhouse environments over three years. In 2019, it was isolated from the final carcasses. In 2020, it was isolated from the final carcasses and chilling water during immersion chilling. In 2021, it was isolated from the final carcasses and surfaces of the grading and packaging workstation. Among the final carcasses, an average occurrence of 40% *L. monocytogenes* was observed, whereas in the environmental samples, it was isolated only from two samples within the clean zone. The occurrence of *L. monocytogenes* in the final carcasses previously ranged between 11.4 and 41.0% (Cox et al., 1997; Rørvik et al., 2003; Sakaridis et al., 2011; Cufaoglu and Ayaz, 2019; Lin et al., 2021). In environmental samples of the slaughterhouse, *L. monocytogenes* was either not detected (Lin et al., 2021) or isolated from various environments, such as cloacal swabs, hands and gloves of workers, door handles of refrigerators, containers holding chicken, workstation surfaces, and cutting boards (Barbalho et al., 2005; Sakaridis et al., 2011; Hosseinzadeh et al., 2012). Cross-contamination can occur at all stages of poultry processing, however, chilling water has been identified as a significant source of contamination (Chiarini et al., 2009), particularly if not strictly controlled, allowing for the proliferation of psychrophilic microorganisms such as *L. monocytogenes*. Our study also confirmed through MLST analysis that most final carcasses and chilling water isolates belonged to ST3, indicating cross-contamination among carcasses during chilling. The high prevalence of *L. monocytogenes* in post-chilling carcasses may be attributed to cross-contamination during chilling (Lin et al., 2021). Additionally, our study revealed that the strains isolated from the final carcasses over three years were primarily ST3, indicating the persistence of these strains within the facility for several years.

However, using culture-dependent methods alone had limitations in determining the points in the facility where cross-contamination occurred and identifying the sources of *L. monocytogenes* contamination. This is primarily due to the challenges in isolating *L. monocytogenes*, such as the sample states and competing organisms. To overcome these limitations, we employed culture-independent methods, such as qPCR and 16S rRNA gene amplicon sequencing, to identify the sources of *L. monocytogenes* contamination along the slaughter line and gather comprehensive information on the whole bacterial communities to assess the linkage between carcass contamination and environmental attributes.

Notably, qPCR detected the presence of *L. monocytogenes* in all samples except for carcasses after scalding, prewashed carcasses after evisceration, and environmental samples in the bleeding area. Compared to the results of *L. monocytogenes* isolation using a culture-dependent method, qPCR was able to identify contamination points of *L. monocytogenes* in a detailed manner. The finding aligns with the recommendation by Moura et al. (2019) to use qPCR in conjunction with culture methods for the confirmation analysis of *L. monocytogenes*, as microbiological methods may yield false-negative results. Considering slaughterhouse’s complex environments, we recommend combining these two methods. The qPCR results (without enrichment) revealed high contamination levels in samples related to live birds, such as feces from truck crates and shackles holding live chickens. Furthermore, qPCR (with enrichment) detected *L. monocytogenes* mostly in the carcass and environmental samples during the final slaughtering stage. These findings are consistent with the isolation of *L. monocytogenes* from chilling water, grading and packaging workstations, and final carcasses using a culture-dependent method. We identified two potential pathways of *L. monocytogenes* contamination from qPCR results. First, based on the qPCR results from non-enriched samples, particularly in feces where *L. monocytogenes* was detected at high levels, asymptomatic chickens carrying *L. monocytogenes* may introduce the bacteria into the facility when they enter slaughterhouses from the farm. Second, based on the qPCR results from enriched samples, *L. monocytogenes* was detected in the samples from the clean zone. This suggests that during the final slaughtering stage, where the chilling temperature is maintained, psychrotrophic *L. monocytogenes* may persist owing to inadequate cleaning, workers’ contaminated gloves, or contaminated workstation surfaces, resulting in cross-contamination of carcasses.

The 16S rRNA gene amplicon sequencing analysis revealed that Proteobacteria and Firmicutes were the dominant phyla in the samples collected from the slaughterhouse, which is consistent with the results of previous studies (Rothrock et al., 2016; Handley et al., 2018; Wages et al., 2019; Wang et al., 2019; Chen et al., 2020a). Notably, the relative abundance of Proteobacteria in the environmental and carcass samples just before immersion chilling (evisceration step) was found to be 84.50–90.12%, accounting for the majority, while it was approximately 40.4% in the chilling water samples. The decrease in abundance of this bacterium aligns with a study that assessed the microbiome in chiller tank water of a poultry slaughterhouse (Rothrock et al., 2016), indicating the impact of chlorine in chilling water on Gram-negative bacteria. By contrast, Firmicutes, including chlorine-resistant Gram-positive bacteria, showed relatively low abundance (4.8–6.1%) in the environmental and carcass samples during evisceration, which increased to 52% in the chilling water samples. At the genus level, the bacterial communities in all samples were predominantly composed of *Acinetobacter*, *Psychrobacter*, and *Pseudomonas,* which have previously been reported to be highly abundant in carcasses and environments of slaughterhouses (Handley et al., 2018; Wang et al., 2019; Chen et al., 2020b). *Acinetobacter* is widely recognized as a dominant microorganism in poultry and meat-processing environments (Lupo et al., 2014; Stellato et al., 2016). In this study, *Acinetobacter* showed the highest relative abundance throughout the slaughter process, particularly with a relative abundance of 81.97% observed on the gloves of evisceration workers. *Acinetobacter* was also detected at high levels in other samples from the evisceration- (27.54–62.19%) and final-stage samples (22.75–36.62%), indicating cross-contamination from evisceration to the final slaughtering stage. *Psychrobacter* and *Pseudomonas* were most abundant in samples following immersion chilling. Specifically, *Pseudomonas* was not detected before immersion chilling, suggesting cross-contamination during chilling, which led to the contamination of the final carcasses. Interestingly, *Pseudomonas* exhibited the highest relative abundance of 32.26% in the chilling water, consistent with the findings of Chen et al. (2020a) and Escudero-Gilete et al. (2005), who concluded that *Pseudomonas* is least affected by the washing process. These results demonstrate that *Acinetobacter* and *Pseudomonas*, known for their psychro-tolerant nature, are more resilient than other organisms to cold-water rinsing and low temperatures of the clean zone (< 10°C). Moreover, since they are spoilage microorganisms that can affect the quality of meat products (Rothrock et al., 2016), targeting these bacteria through hygiene management in the clean zone is crucial during the final slaughtering stage.

Based on alpha diversity, we observed a general decrease in species richness along the slaughter line (*p* < 0.001), suggesting the effectiveness of each hurdle in reducing bacterial populations during slaughtering. However, we noted a transient increase in species richness in the prewashed carcasses after evisceration (*p* < 0.05). This can be attributed to cross-contamination of the carcasses by the gut microbiome during evisceration. By contrast, the species diversity of the carcasses significantly decreased during scalding (*p* < 0.001), indicating that only thermophilic bacteria capable of surviving at high temperatures were present on the carcasses after being scalded in hot water. Specifically, the relative abundances of thermophilic bacteria, *Anoxybacillus* and *Ureibacillus*, significantly increased in the carcasses after scalding compared with those before scalding (*Anoxybacillus*, 0.03–39.83%; *Ureibacillus*, 0.02–39.67%), and they accounted for the highest proportion in the bacterial composition of the carcasses after scalding. Subsequently, species diversity significantly increased during defeathering and evisceration, indicating the occurrence of cross-contamination by diverse bacterial species during these two stages. Furthermore, evenness significantly decreased during the final washing stage (*p* < 0.001), suggesting an overall reduction in bacterial species and survival of psychrotrophic bacteria because of rinsing with cold water. In fact, the relative abundance of psychro-tolerant *Acinetobacter* increased after washing and accounted for the highest proportion (27.54–42.93%). Therefore, we could identify scalding, defeathering, evisceration, and washing as critical stages in altering the microbial structure, emphasizing defeathering and evisceration as significant reasons for cross-contamination. The PCoA clustering results showed that the final carcasses shared a relatively high number of taxa with the grading and packaging workstation and walls. This beta diversity suggests that a clean zone environment is essential for controlling the nature of bacterial communities originating from the final carcasses.

We then applied the SourceTracker modeling using a Bayesian algorithm to estimate the proportion of each source contributing to a designated sink sample and traced the transmission routes of these microorganisms. Based on the environmental samples (sources) taken from three zones (contaminated, semi-clean, and clean zone) of the slaughterhouse, bacteria from the sources were primarily associated with carcass samples (sinks) collected from the same zone. However, the microbial communities of shackles and chilling water were linked to carcass samples in different zones, extending beyond their respective zones. Considering that shackles move along the rail between the contaminated and semi-clean zones, we speculated that the shackles from the contaminated zone cross-contaminated the carcass samples in the semi-clean zone. Furthermore, the microbial community in the chilling water was associated with the carcass samples from all zones. This indicates that bacteria from the carcass samples before chilling contaminated the chilling water and formed the microbial community. This bidirectional source/sink relationship was evident in the bacterial flow during the source tracking analysis. Thus, the microbial community present in the chilling water can be linked to the final carcasses in the clean zone. In congruence with the clustering observed in beta diversity, the source tracking results indicated that the microorganisms in the final carcasses were significantly correlated with those on the surface of the grading and packaging workstation, followed by those of the walls in the grading and packaging area and chilling water. The grading and packaging workstation was the final contact point with carcasses immediately before exiting the slaughterhouse. This point was already the last section of the clean zone, and no interventions were observed throughout the study. Therefore, if this section is contaminated with bacteria that cause spoilage or foodborne diseases, the final carcasses may inevitably become cross-contaminated. The wall surface of the clean zone, which was the second source of contamination of the final carcasses, was a non-contact point of carcasses; however, it could be contaminated by aerosolized bacteria. Therefore, after slaughtering, the clean zone’s non-contact and contact areas must be cleaned. The third cause of carcass contamination was the chilling water, which has been previously identified as a potential source of contamination (Göksoy et al., 2004; Guerin et al., 2010; Wang et al., 2019). Since broilers are moved to the grading and packaging zone after immersion chilling, any bacteria remaining on carcasses may cause cross-contamination while handling and packaging all products that follow through to the end of processing. Furthermore, this can lead to shelf-life issues and affect consumers.

Taken together, microbiological and 16S rRNA gene amplicon sequencing analyses demonstrate that contamination of the final carcasses is mainly affected by immersion chilling and subsequent steps. Furthermore, we have provided evidence that specific environments during slaughtering impact the composition of microbial communities. Specifically, the proliferation of psychro-tolerant bacteria, such as *L. monocytogenes* and *Pseudomonas*, in the immersion chilling tank and clean zone where the final carcasses are handled is a significant issue.

## 5 Conclusion

*L. monocytogenes* was consistently isolated in the slaughterhouse for three years, indicating its persistence and ability to contaminate chicken carcasses during slaughtering. We investigated the sequential transmission of *L. monocytogenes* along the processing line by isolating identical STs from final carcasses and chilling water using traditional culture-dependent methods. Results of culture-independent methods revealed widespread contamination of *L. monocytogenes* in the slaughterhouse. 16S rRNA gene amplicon sequencing analysis suggested that the environmental characteristics of the clean zone favored the proliferation of psychro-tolerant bacteria. Importantly, metagenomics-based microbial source tracking identified environmental sources influencing carcass contamination, with chilling and subsequent steps in the clean zone playing a significant role. The combined use of culture-independent and -dependent methods provided a comprehensive understanding of the bacterial transmission routes and microbial community dynamics in the slaughterhouse environment.

## Data availability statement

The data presented in the study are deposited in the NCBI sequence read archive repository, with bioproject accession no. PRJNA971784.

## Author contributions

JJ: Conceptualization, Data curation, Formal analysis, Investigation, Methodology, Software, Visualization, Writing – original draft, Writing – review & editing. HS: Software, Supervision, Validation, Visualization, Writing – review & editing. W-HK: Supervision, Validation, Writing – review & editing. MC: Project administration, Resources, Writing – review & editing. J-YL: Funding acquisition, Project administration, Writing – review & editing. Y-KK: Funding acquisition, Project administration, Resources, Writing – review & editing. SC: Conceptualization, Supervision, Validation, Writing – review & editing.

## Footnotes

^1^ https://foodsafetykorea.go.kr/foodcode/01_03.jsp?idx=386

^2^ https://bigsdb.pasteur.fr/listeria/primers-used/

^3^ https://www.ezbiocloud.net

^4^ https://github.com/danknights/sourcetracker
