# Supplementary material for: Tracking the contamination sources of microbial population and characterizing Listeria monocytogenes in a chicken slaughterhouse by using culture-dependent and -independent methods
Source: Front Microbiol. 2023 Nov 30;14:1282961. doi: 10.3389/fmicb.2023.1282961 (PMC10720907; doi:10.3389/fmicb.2023.1282961)
Supplement: Supplementary file 1 [file Table_1.DOCX]

Supplementary Material

Tracking the Contamination sources of Microbial Population and Characterizing *Listeria monocytogenes* in a Chicken Slaughterhouse by Using Culture-Dependent and -Independent Methods

Jiyeon Jeong, Hyokeun Song, Woo-Hyun Kim, Myeongju Chae, Ji-Youn Lee, Yong-Kuk Kwon and Seongbeom Cho^*^

*** Correspondence:** Seongbeom Cho: [chose@snu.ac.kr](mailto:chose@snu.ac.kr)

# Supplementary Figures and Tables

## Supplementary Tables

**Supplementary Table 1.** Processing characteristics of the slaughterhouse

| No. of Line | 1 |
| --- | --- |
| Production capacity (chickens/day) | 60,000 |
| Line speed (chickens/hour) | 7,500 |
| No. of scalding tank | 1 |
| Minimum scalding temperature (^o^C) | 59.0 |
| Maximum scalding temperature (^o^C) | 59.5 |
| Scalding time | 2 min 30 s |
| Plucking time | 35 s |
| Water-immersion chilling sanitizer | Chlorinated water (20~50 ppm) |
| Type of immersion water chiller | Counterflow |
| Immersion water chiller tank temperature (^o^C) | 5 |
| Time in immersion water chiller | 50 min |
| Water turnover (L/min) | 15 |
