# Supplementary material for: Tracking the contamination sources of microbial population and characterizing Listeria monocytogenes in a chicken slaughterhouse by using culture-dependent and -independent methods
Source: Front Microbiol. 2023 Nov 30;14:1282961. doi: 10.3389/fmicb.2023.1282961 (PMC10720907; doi:10.3389/fmicb.2023.1282961)
Supplement: Supplementary file 2 [file Table_2.DOCX]

Supplementary Material

Tracking the Contamination sources of Microbial Population and Characterizing *Listeria monocytogenes* in a Chicken Slaughterhouse by Using Culture-Dependent and -Independent Methods

Jiyeon Jeong, Hyokeun Song, Woo-Hyun Kim, Myeongju Chae, Ji-Youn Lee, Yong-Kuk Kwon and Seongbeom Cho^*^

*** Correspondence:** Seongbeom Cho: [chose@snu.ac.kr](mailto:chose@snu.ac.kr)

# Supplementary Figures and Tables

## Supplementary Tables

**Supplementary Table 2.** *Listeria monocytogenes* isolates for microbiological analysis

| Strain ID | Isolation date | Source |
| --- | --- | --- |
| 19-FC-4-LM | August 27, 2019 | Carcass after immersion chilling^a^ |
| 19-FC-5-LM | August 27, 2019 | Carcass after immersion chilling |
| 19-FC-6-LM | August 27, 2019 | Carcass after immersion chilling |
| 19-FC-7-LM | August 27, 2019 | Carcass after immersion chilling |
| 19-FC-8-LM | August 27, 2019 | Carcass after immersion chilling |
| 19-FC-9-LM | August 27, 2019 | Carcass after immersion chilling |
| 20-FC-6-LM | August 11, 2020 | Carcass after immersion chilling |
| 20-FC-7-LM | August 11, 2020 | Carcass after immersion chilling |
| 20-CW-22-LM | August 11, 2020 | Chilling water |
| 21-FC-2-LM | September 4, 2021^b^ | Carcass after immersion chilling |
| 21-FC-4-LM | September 4, 2021 | Carcass after immersion chilling |
| 21-FC-9-LM | September 4, 2021 | Carcass after immersion chilling |
| 21-GP-61-LM | September 4, 2021 | Workstation surface of grading and packaging |

^a^ Final carcass

^b^ Samples collected in this study
