# Supplementary material for: Tracking the contamination sources of microbial population and characterizing Listeria monocytogenes in a chicken slaughterhouse by using culture-dependent and -independent methods
Source: Front Microbiol. 2023 Nov 30;14:1282961. doi: 10.3389/fmicb.2023.1282961 (PMC10720907; doi:10.3389/fmicb.2023.1282961)
Supplement: Supplementary file 3 [file Table_3.DOCX]

Supplementary Material

Tracking the Contamination sources of Microbial Population and Characterizing *Listeria monocytogenes* in a Chicken Slaughterhouse by Using Culture-Dependent and -Independent Methods

Jiyeon Jeong, Hyokeun Song, Woo-Hyun Kim, Myeongju Chae, Ji-Youn Lee, Yong-Kuk Kwon and Seongbeom Cho^*^

*** Correspondence:** Seongbeom Cho: [chose@snu.ac.kr](mailto:chose@snu.ac.kr)

# Supplementary Figures and Tables

## Supplementary Tables

**Supplementary Table 3.** Positive rates of *Listeria monocytogenes* from different sampling sources collected along the slaughter line

|  |  |  | **2019** | | **2020** | | **2021** | |
| --- | --- | --- | --- | --- | --- | --- | --- | --- |
| **Sampling site** | **Slaughter step** | **Source** | **No. of Samples** | **Positive No. of Samples (%)** | **No. of Samples** | **Positive No. of Samples (%)** | **No. of Samples** | **Positive No. of Samples (%)** |
| Contaminated  zone | Entry | Feces from crates | NS | NA | 5 | 0 (0.0) | 5 | 0 (0.0) |
|  | Hanging | Shackles | NS | NA | 5 | 0 (0.0) | 5 | 0 (0.0) |
|  | Bleeding | Wall | NS | NA | NS | NA | 3 | 0 (0.0) |
|  |  | Floor | NS | NA | NS | NA | 3 | 0 (0.0) |
| Semi-clean zone | Scalding | Carcasses before scalding | NS | NA | NS | NA | 3 | 0 (0.0) |
|  |  | Carcasses after scalding | NS | NA | NS | NA | 3 | 0 (0.0) |
|  | Defeathering | Feathers | NS | NA | NS | NA | 5 | 0 (0.0) |
|  |  | Carcasses after defeathering | NS | NA | NS | NA | 3 | 0 (0.0) |
|  | Evisceration | Gloves | NS | NA | NS | NA | 5 | 0 (0.0) |
|  |  | Workstation | NS | NA | NS | NA | 5 | 0 (0.0) |
|  |  | Carcasses after evisceration | NS | NA | NS | NA | 3 | 0 (0.0) |
|  |  | Carcasses after washing | NS | NA | NS | NA | 3 | 0 (0.0) |
| Clean zone | Immersion chilling | Chilling water | NS | NA | **3** | **1 (33.3)** | 3 | 0 (0.0) |
|  | Grading and  packaging | Wall | NS | NA | NS | NA | 3 | 0 (0.0) |
|  |  | Workstation | NS | NA | NS | NA | **3** | **1 (33.3)** |
|  |  | Final carcasses | **10** | **6 (60.0)** | **10** | **2 (20.0)** | **10** | **3 (30.0)** |
| **Total** |  |  | **10** | **6 (60.0)** | **23** | **3 (13.0)** | **65** | **4 (6.2)** |

NS: non-sampling, NA: not available
