# Supplementary material for: Tracking the contamination sources of microbial population and characterizing Listeria monocytogenes in a chicken slaughterhouse by using culture-dependent and -independent methods
Source: Front Microbiol. 2023 Nov 30;14:1282961. doi: 10.3389/fmicb.2023.1282961 (PMC10720907; doi:10.3389/fmicb.2023.1282961)
Supplement: Supplementary file 4 [file Table_4.DOCX]

Supplementary Material

Tracking the Contamination sources of Microbial Population and Characterizing *Listeria monocytogenes* in a Chicken Slaughterhouse by Using Culture-Dependent and -Independent Methods

Jiyeon Jeong, Hyokeun Song, Woo-Hyun Kim, Myeongju Chae, Ji-Youn Lee, Yong-Kuk Kwon and Seongbeom Cho^*^

*** Correspondence:** Seongbeom Cho: [chose@snu.ac.kr](mailto:chose@snu.ac.kr)

# Supplementary Figures and Tables

## Supplementary Tables

**Supplementary Table 4.** Detection of *Listeria monocytogenes* in chicken slaughterhouse samples according to cultural and qPCR methods

| **Sampling site** | **Slaughter step** | **Source** | **Microbiological**  **analysis** | **qPCR of *prfA* gene** | | |
| --- | --- | --- | --- | --- | --- | --- |
|  |  |  |  | **Raw samples** | **Enrichment samples  (LEB)** | **Enrichment samples (Fraser)** |
| Contaminated  zone | Entry | Feces from crates | - | + (7.34 ± 0.17)* | + (3.68 ± 0.04) | + (3.15 ± 0.02) |
|  | Hanging | Shackles | - | + (2.13 ± 0.4) | - | + (4.63 ± 0.03) |
|  | Bleeding | Wall | - | - | - | - |
|  |  | Floor | - | - | - | - |
| Semi-clean zone | Scalding | Carcasses before scalding | - | - | + (5.52 ± 0.15) | - |
|  |  | Carcasses after scalding | - | - | - | - |
|  | Defeathering | Feathers | - | - | + (0.61 ± 0.03) | + (0.97 ± 0.01) |
|  |  | Carcasses after defeathering | - | - | - | + (2.44 ± 0.03) |
|  | Evisceration | Gloves | - | - | - | + (3.11 ± 0.02) |
|  |  | Workstation | - | - | + (0.22 ± 0.08) | + (1.57 ± 0.01) |
|  |  | Carcasses after evisceration | - | - | - | - |
|  |  | Carcasses after washing | - | - | - | + (1.92 ± 0.01) |
| Clean zone | Immersion chilling | Chilling water | - | - | + (3.79 ± 0.04) | NS |
|  | Grading and packaging | Wall | - | - | - | - |
|  |  | Workstation | + | - | - | + (4.24 ± 0.01) |
|  |  | Final carcasses | + | - | + (3.90 ± 0.03) | + (4.04 ± 0.01) |

NS: non-sampling, these samples were considered negative as there was no color change in the Fraser broth, and therefore, they were not used as qPCR samples.
* mean ± SEM log CFU/mL
