# Supplementary material for: Tracking the contamination sources of microbial population and characterizing Listeria monocytogenes in a chicken slaughterhouse by using culture-dependent and -independent methods
Source: Front Microbiol. 2023 Nov 30;14:1282961. doi: 10.3389/fmicb.2023.1282961 (PMC10720907; doi:10.3389/fmicb.2023.1282961)
Supplement: Supplementary file 5 [file Table_5.DOCX]

Supplementary Material

Tracking the Contamination sources of Microbial Population and Characterizing *Listeria monocytogenes* in a Chicken Slaughterhouse by Using Culture-Dependent and -Independent Methods

Jiyeon Jeong, Hyokeun Song, Woo-Hyun Kim, Myeongju Chae, Ji-Youn Lee, Yong-Kuk Kwon and Seongbeom Cho^*^

*** Correspondence:** Seongbeom Cho: [chose@snu.ac.kr](mailto:chose@snu.ac.kr)

# Supplementary Figures and Tables

## Supplementary Tables

**Supplementary Table 5.** Relative abundance (%) of the four most abundant phyla

| **Sample groups** | **Sampling point** | ***Proteobacteria*** | ***Firmicutes*** | ***Actinobacteria*** | ***Bacteroidetes*** | **Others^a^** |
| --- | --- | --- | --- | --- | --- | --- |
| Environment | Feces | 44.78 | 51.24 | 3.60 | 0.32 | 0.06 |
|  | Shackles | 68.28 | 28.97 | 2.22 | 0.00 | 0.53 |
|  | Bleeding_floor | 12.07 | 28.30 | 56.54 | 2.57 | 0.51 |
|  | Bleeding_wall | 5.38 | 70.56 | 23.95 | 0.00 | 0.10 |
|  | Feathers | 46.15 | 48.87 | 1.33 | 3.13 | 0.53 |
|  | Evisceration desk | 84.50 | 4.83 | 1.55 | 9.05 | 0.06 |
|  | Evisceration_gloves | 84.62 | 0.00 | 13.10 | 1.32 | 0.97 |
|  | Chilling water | 40.44 | 52.02 | 1.53 | 5.25 | 0.76 |
|  | Clean zone_ workstation | 66.19 | 3.10 | 14.72 | 10.85 | 5.13 |
|  | Clean zone_wall | 72.60 | 1.77 | 18.76 | 6.08 | 0.79 |
| Carcass | Before scalding | 13.84 | 78.34 | 6.10 | 1.60 | 0.13 |
|  | After scalding | 3.08 | 94.23 | 1.77 | 0.00 | 0.92 |
|  | After defeathering | 80.47 | 11.87 | 2.81 | 4.80 | 0.05 |
|  | Before washing | 66.32 | 26.10 | 2.25 | 5.22 | 0.12 |
|  | After washing | 90.12 | 6.11 | 1.34 | 2.42 | 0.01 |
|  | Final carcasses | 79.66 | 13.24 | 3.47 | 3.55 | 0.08 |

^a^ 40 phyla, relative abundance of < 1%
